# When "In Your Face" Is Not Out of Place: The Effect of Timing of Disclosure of a Same-Sex Dating Partner under Conditions of Contact

**DOI:** 10.1371/journal.pone.0135023

**Published:** 2015-08-26

**Authors:** Sharon K. Dane, Barbara M. Masser, Geoff MacDonald, Julie M. Duck

**Affiliations:** 1 School of Psychology, McElwain Building, The University of Queensland, St. Lucia, QLD 4072, Australia; 2 Department of Psychology, Sidney Smith Bldg, University of Toronto, 100 St. George Street, Toronto, Ontario, M5S 3G3, Canada; 3 Faculty of Humanities and Social Sciences, Forgan Smith Bldg, The University of Queensland, St. Lucia, QLD 4072, Australia; The University of Texas at Austin, UNITED STATES

## Abstract

In a series of experiments we examined heterosexuals’ reactions to the timing of disclosure of a gender-matched confederate’s same-sex dating partner. Disclosure occurred in a naturalistic context–that is, it occurred when meeting, or expecting to soon meet, a same-sex attracted individual, who voluntarily shared this information with the participant as a natural part of a broader topic of discussion. The confederate, when disclosing early rather than later, was approached more closely (Prestudy) and liked more (Studies 1–2). Those experiencing early disclosure, compared with later, were less drawn to topics of lower intimacy (Study 1), were happier and more excited about meeting the confederate, and more likely to choose to be alone with the confederate for a one-on-one discussion (Study 2). Further, women experiencing early disclosure were more willing to introduce the same-gender confederate to their friends (Study 2). The benefits of knowing sooner, rather than later, continued to apply even when participants were given further time to process the disclosure. To explore the underlying reasons for the more favorable experiences of upfront disclosure, we examined participants’ memory of the information shared by the confederate (Study 3). Results revealed that those who experienced delayed disclosure were more likely to incorrectly recall and negatively embellish information related to the confederate’s sexual orientation, suggesting that early disclosure resulted in a reduced tendency to focus on the confederate’s sexuality as a defining feature. These positive findings for early timing are discussed in light of previous studies that have found benefits for delayed disclosure and those that have failed to investigate the effects of timing of ‘coming out’ under conditions of contact.

## Introduction

Consistent with the contact hypothesis [1], meta-analyses show that contact between members of different groups can reduce intergroup prejudice [2–4]. While negative contact, with threat perceived from outgroup members particularly in relation to a history of intergroup conflict, predicts higher levels of prejudice [4–6], positive forms of contact can yield benefit for intergroup relations. Positive contact, at least from the perspective of majority group members, is shown to typically improve attitudes toward a stigmatized minority [4, 7]. Researchers characterize optimal contact as contact comprising experiences that lead to intergroup friendship [4, 8–10], with the potential to develop these friendships added as a key facilitating condition of the contact hypothesis [9].

Findings from experimental laboratory [11, 12] and experimental field research [13] highlight the importance of developing cross-group friendships to increase the chances of favorable attitudes generalizing to the outgroup as a whole. Even indirect cross-group friendships (i.e. just knowing that an ingroup friend has an outgroup friend) can generalize to more benevolent group judgements [14, 15]. While recent evidence suggest that the impact of this extended contact on attitudinal change may be less stable than direct contact in the short term [4, 16], it can be beneficial when certain groups have limited access to outgroup members or when there is a desire to avoid intergroup contact.

Whether intergroup friendships are direct or indirect, however, the benefits of these relationships hinge on at least some individuals forming close ties with outgroup members and there being an awareness of each other’s group status. That is, intergroup contact cannot actually be perceived as being ‘intergroup’ (and therefore beneficial in terms of intergroup relations) unless individuals are aware of their different group memberships. In the case of a potentially concealable and stigmatized group membership, such as a same-sex sexual orientation, this presents a dilemma. Several contact studies have already shown that cross-group friendships with same-sex attracted individuals (i.e., those whose sexual orientation has already become apparent to the heterosexual individual) is associated with more positive heterosexual attitudes toward this minority group in general [17–19]. What is unclear, however, is whether upfront or delayed self-disclosure of such an outgroup status is most likely to have allowed these cross-group friendships to have developed in the first place. Studies that have examined outgroup members’ reactions to the timing of disclosure of a person’s same-sex sexual orientation have varied significantly in methodological approach, with empirical evidence provided in support of both upfront [20, 21] and delayed disclosure [22–24]. The purpose of the current research is to further test the impact of the timing of this disclosure under conditions we argue are perhaps more likely to reflect how same-sex attracted individuals may choose to convey this information during their everyday interactions with others. Under conditions of contact and anticipated contact, we examine the experience from the heterosexual (i.e. majority group) perspective. We begin by presenting research on self-disclosure during intergroup contact and the timing of self-disclosure more broadly, followed by the mixed findings on the effect of timing of disclosure of a same-sex sexual orientation.

### Self-disclosure during intergroup contact

Studies have highlighted the importance of inducing positive affect during intergroup encounters, particularly through the role of self-disclosure [25, 26]. In a series of studies, Turner, Hewstone, and Voci [27] found that the positive relationship between cross-group friendships and outgroup attitudes was mediated by reciprocal self-disclosure. Further, the positive relationship between self-disclosure and outgroup attitudes was explained by an increase in empathy, intergroup trust, and the perceived value of intergroup contact. The authors conclude that the inclusion of self-disclosure should be a key component of social interventions aimed at reducing prejudice, with this supported in a recent meta-analytic review of studies on cross-group friendships and outgroup attitudes [8].

Most research involving intergroup contact, however, has related to racial/ethnic groups in which ingroup members’ awareness of the other person’s outgroup status is immediate and occurs prior to other aspects of self-disclosure. In fact, Miller [28] notes that contact at the interpersonal level usually occurs in the presence of category-identifying information, such as age, skin color, facial features, and linguistic cues. With outgroup status already established self-disclosure can then focus on other defining aspects of an individual. In the case of a concealable group membership, however, contact may lead to the realization that someone initially perceived to be an ingroup member is in fact an outgroup member. Therefore, even when conditions of contact allow for interpersonal closeness (i.e., self-disclosure in general) there remains the important question of whether a sexual minority individual will receive more positive reactions if they self-disclose their sexual minority status upfront, or after a period of self-disclosure on other topics.

### Timing of self-disclosure

Research on impression formation provides evidence for the benefit of both early and delayed self-disclosure. In support of delayed disclosure, research on the primacy effect has suggested that information presented early during impression formation often dominates the overall evaluation of the individual [29, 30]. Thus, if same-sex attraction is perceived as somewhat negative, early disclosure may tarnish any forthcoming positive information. However, research on the recency effect has suggested that when information is perceived to be very negative or deviate greatly from a positive norm, this late information will dominate perceptions and preceding information will be ignored [31]. In this case, delaying disclosure could result in less favorable evaluations. Early attribution theory studies also provide support for both stages of timing. Although people generally react more positively to delayed disclosure of negative information [32, 33], early disclosure of negative information is beneficial provided the discloser volunteers the information and someone else is not to blame for the negative outcome [32–34]. In this instance, the more positive findings for early disclosure were attributed to the perceived virtue of an upfront truthful confession. In fact, research shows that reactions to the disclosure of a stigmatized status in general may vary depending on the nature of the stigma, not only in terms of being responsible for the stigma, but the person’s control over it, its concealability, and its perceived threat to the recipient of the disclosure [35]. This suggests that the effects of timing of disclosure may also be dependent on the type of stigma disclosed.

#### Timing of disclosure of a same-sex sexual orientation

The limited research that has specifically investigated the timing of disclosure of a same-sex sexual orientation has yielded mixed results, which may not be surprising given the wide variety of methodological approaches employed. In Golebiowska’s study [23], sexual orientation disclosure occurred indirectly in a written description of a political candidate. The results showed that participants evaluated the candidate more positively when the description involved delayed disclosure rather than early. Consistent with this, King and colleagues [24] found that when participants were asked to imagine a scenario in which a fictitious gay co-worker ‘came out’ early (within one week) or two years down the track, responses were more positive among those who imagined the delayed ‘two years later’ scenario. Buck and Plant [22] extended upon these earlier experiments by including anticipated contact with a same-sex attracted individual. Participants experienced a male confederate’s disclosure of his sexual orientation early or late during a scripted interview (in the first study via audio recording and in the second via video). Participants were led to believe they would soon meet the confederate. In both studies male participants reacted more favorably to delayed disclosure relative to early disclosure. Female participants’ reactions to the disclosure were similar regardless of the timing. These findings for the benefits of delaying ‘coming out’ regarding one’s sexual orientation, at least to men, are in keeping with the results of Golebiowska [23] and King et al. [24].

Other studies, however, have found benefits for upfront disclosure. In an early study by Gross and colleagues [20], participants were exposed to a gay or lesbian confederate’s disclosure of their sexual orientation before or after getting to know them in a 3-minute videoed interview. Findings showed that early disclosure, compared with delayed, resulted in greater liking of the gay and lesbian confederate but only by male participants. MacInnis and Hodson [21] tested the effect of timing of disclosure of sexual orientation while participants interacted with an ostensible online partner, during a task designed to induce interpersonal closeness. Early disclosure resulted in more positive reactions from both male and female participants.

Although the varied methodologies used in these studies make comparing them difficult, there are notable limitations in each case. For example, with the exception of Buck and Plant’s [22] research, which found benefits for delayed disclosure, none of the studies involved the prospect of actually meeting the same-sex attracted individual. Although research shows that expecting to meet a stigmatised outgroup member can result in people displaying behavior associated with high levels of anxiety [36], close contact with minority group members is critical for cross-group friendships to occur, which can assist in improving intergroup relations [8–10]. Further, none of the studies involved the same-sex attracted individual disclosing directly to the participant. Instead participants experienced the disclosure either (a) indirectly through witnessing the confederate ‘come out’ to an interviewer [22], (b) by reading a profile of the individual, in which sexual orientation was provided as a consequence of required or requested information [21, 23], (c) by being asked to imagine the person disclosing to them [24] or (d) by being privy to information provided in a questionnaire [20].

The fact that direct disclosure was not tested in any of these studies is likely to be a significant caveat. Two-wave data (from one year to the next) from a US national telephone survey found that the vast majority of heterosexual individuals with a gay or lesbian friend reported being told directly by the person about his or her sexuality [17]. Further, those who experienced direct disclosure had more favorable attitudes towards sexual minorities than those who had found out by another means. The results of the cross-wave analyses suggested that while pre-existing positive attitudes may attract such disclosure, such experiences are also likely to improve attitudes. This is in keeping with social penetration theory [37], which posits that people tend to feel greater liking towards others who voluntarily disclose to them. As shown in earlier attribution studies [32, 34], it may be something about the personal and voluntary nature of such disclosure that makes positive reactions from the recipients of early disclosure more likely.

Further, if such information is to be voluntarily conveyed by a same-sex attracted individual then how this is communicated to another may also be critical, given upfront disclosure would take place when very little, if anything, is known about the other. In studies that found less favorable reactions to upfront disclosure the disclosure was overt. For example, in King et al. [24] early disclosure condition participants were asked to imagine a colleague telling them “I am gay”. In Buck and Plant [22] participants witnessed a confederate tell an interviewer that he had a boyfriend when simply asked if he was single or in a relationship. Although there may be circumstances under which a person chooses to disclose in such a manner, explicitly focusing on a person’s sexuality during the very initial stages of getting to know someone may be considered by some to be “too much too soon”. That is, upfront disclosure of what may be perceived to be highly personal information could be deemed inappropriate and a violation of social norms [37, 38].

Disclosure of a person’s sexual orientation, however, need not be explicit. As Herek [39] pointed out, heterosexual individuals disclose their relationship status on a regular basis and as a natural part of their everyday interactions with others. For example, a woman may simply make the comment “I didn’t bring the car to work today because my husband will be picking me up”. Although it becomes clear that this person is in a relationship with someone of a different sex, it is not the topic of discussion. Research shows that same-sex attracted individuals can, and do, disclose their sexuality in much the same way. In a nation-wide survey of over 2000 same-sex attracted Australians, the majority responded that they would ‘come out’ early when presented with various social gathering (i.e., non-work related) scenarios but only if the disclosure was relevant to the conversation [40]. When asked to provide examples, one woman explained “I don’t yell, ‘Hi, I’m in a relationship with a woman!’ I speak normally and use the correct pronouns”. A male participant explained “I would try and make it as similar and as casual as if I were introducing my girlfriend, if I was straight”. These comments, and hundreds like them in the survey, show that upfront disclosure for same-sex attracted individuals can be a natural part of their everyday self-expression in much the same way heterosexuals are able to convey who they are in terms of their relationships with others. Importantly, being able to integrate same-sex sexual orientation disclosure naturally into a relevant topic of conversation may be less likely to be perceived as a violation of norms, particularly during interactions involving strangers. In a sense, the disclosure is normalized by embedding it in the details of everyday life.

The aim of the current research, therefore, is to address the discrepancy found in previous studies by examining the impact of timing of disclosure of a same-sex sexual orientation under arguably more natural conditions of contact. That is, disclosure occurs when meeting, or expecting to soon meet, a same-sex attracted individual, who voluntarily shares this information with the participant as a natural part of a broader topic of discussion. As a number of idiosyncratic aspects of methodology may influence the effect of timing of disclosure, the aim of this research is not to directly test the current approach against the varied approaches of previous studies. Instead, we examine the impact of disclosure timing under conditions that are perhaps more likely to reflect how same-sex attracted individuals can choose to convey the nature of their relationships through their everyday interactions with others [40]. In employing a more personal and perhaps less confronting approach, we predict that ‘coming out’ sooner, rather than later, will result in more favorable reactions from recipients of the disclosure. In this context, and in contrast to Buck and Plant [22], we predict more positive reactions to early timing under circumstances that allow participants to experience actual or anticipated contact with a same-sex attracted individual.

As research on promoting positive intergroup attitudes has focused on the importance of generating interpersonal closeness during cross-group interactions [9, 28, 41], the current study tests the effect of timing of ‘coming out’ while participants engage in a broader disclosure task designed to induce interpersonal closeness under experimental conditions. However, studies show that people tend to express greater discomfort when considering contact with a homosexual of their own sex than of a different sex [42–44]. La Mar and Kite [45] suggested that contact may be a distinct component of attitudes towards homosexuality, as their research showed that it was only in relation to contact (i.e., people’s attitudes in relation to having a homosexual colleague or neighbor) that women, as well as men, reported more negative attitudes towards homosexuals of their own sex. Therefore, to improve such intergroup contact, at least from a heterosexual individual’s perceptive, research may benefit from investigating the contact experience for heterosexual men when engaging with same-sex attracted men, and heterosexual women when engaging with same-sex attracted women. Consistent with MacInnis and Hodson’s [21] study on timing of sexual orientation disclosure, we address this issue by pairing participants with a confederate of their own sex.

In our first study (the prestudy), female participants undertook a modified version of the Relationship Closeness Induction Task (RCIT) [46] with a confederate who disclosed having a same-sex dating partner either early or late during a 45-minute face-to-face interaction. To test the findings of the prestudy with a larger sample, and with both male and female participants, Study 1 varied the initial paradigm. Under conditions of anticipated contact, participants experienced a same-gender confederate disclose to them early or late during an exchange of personal information via computer. To test timing of disclosure in relation to whether the disclosure was embedded in a more casual or intimate story, we also manipulated the context of the disclosure. Study 2 replicated Study 1, but allowed additional time for participants to process the disclosure. Finally, Study 3 examined the potential underlying mechanisms for preferences in timing of disclosure by testing participants’ memory (recall) of the information shared by the confederate under the different disclosure conditions.

All participants were undergraduates from a large Australian university. The confederates were from an acting academy, 18–20 years of age, and genuinely same-sex attracted (although, without disclosure, assumed heterosexual in pilot research).

## Prestudy

We first test the efficacy of our methodological approach, using the RCIT, in a small-scale study involving actual contact with a same-sex attracted confederate. We anticipated that actual contact would be more confronting than more distal forms of contact and therefore would be more likely to produce a significant effect for timing of disclosure with a relatively small sample. As heterosexual women generally have more positive attitudes toward same-sex sexuality, compared with heterosexual men [44, 45], our prestudy also involved a more stringent test of the effect of timing of disclosure, through the engagement of female participants who were paired with a female confederate. The disclosure of the confederate’s sexuality occurred either early or late when exchanging personal information during a 45-minute face-to-face interaction. The resulting *social distance* behavior of the participant was assessed.

### Method

#### Ethics Statement

All studies for the current research were approved by the Behavioural and Social Sciences Ethical Review Committee for the University of Queensland, and comply with the provisions contained in the Australian *National Statement on Ethical Conduct in Human Research* and the regulations governing experimentation on humans (clearance numbers: 2004000496, 2012001316). Written consent was obtained in all cases. As the first three experiments involved aspects of deception, participants were fully debriefed immediately after the studies both in writing and in person. During the debriefing, it was made clear that the researchers were not interested in people’s reactions toward heterosexuals vs. sexual minorities. It was explained that the purpose of the research was to investigate participants’ reactions to the different timing of ‘coming out’ as same-sex attracted, for which there was no ‘right’ or ‘wrong’ way to respond.

#### Pre-interaction

Individual participants (20 undergraduate women, mean age = 18.5, *SD* = .83) were instructed that they would exchange personal information both in writing and in conversation with another female participant. The participant and the confederate (acting as another student) entered the same room and first completed Altemeyer’s [47] 12-item Right-Wing Authoritarianism scale (RWA) and Mattick and Clarke’s [48] Social Interaction Anxiety Scale (SIAS). The RWA scale (e.g., “some of the worst people in our country nowadays are those who do not respect our flag, our leaders, and the normal way things are supposed to be done”) was used as an indirect measure of prejudice towards homosexuality, to ensure participants were not alerted to the purpose of the research before debriefing. A meta-analysis conducted by Whitley and Lee [49] found RWA to be a good predictor of attitudes toward lesbians and gay men, even when the homosexual items were not included in the scale, as was the case for the current study. The SIAS consists of 19 items (e.g., “when mixing socially I feel uncomfortable”). It was included to control for participants’ levels of general social interaction anxiety. Response options for the RWA and SIAS scales ranged from 1 = *very strongly disagree* to 9 = *very strongly agree*.

#### Disclosure manipulation

On completion of the above questionnaires and prior to discussion with their task partner, the participant (and the confederate) were instructed to write two brief stories about happy or funny memories involving friends or a dating partner and told that they would later exchange them. The confederate’s stories were pre-written and sealed to ensure that the confederate and experimenter were blind to the experimental conditions. To appear credible, the confederate simply went through the motions of writing the stories.

As the timing of the topic of discussion in which disclosure is embedded may also have an effect, two different stories were used and the order counterbalanced. In one story the confederate wrote about a funny experience when she went to the movies with someone (*disclosure*—this girl) she was dating at the time. She eventually noticed her parents were seated a few rows back and that she remembered feeling silly but that her date (*disclosure*—girlfriend) thought it was hilarious. In the other story the confederate wrote about going to all the trouble of preparing a wonderful romantic dinner at her flat for this really cool person (*disclosure*—girl) she had met. While feeling exhausted and waiting eagerly for the doorbell to ring, she realised when looking at the fridge calendar that the dinner was for the next night.

The confederate either revealed the dating partner’s gender in the first story exchange (movie or dinner—*Early* condition) or in the second story exchange (movie or dinner—*Delayed* condition).

#### Relationship Closeness Induction Task

After the participant and confederate personally exchanged the first of the two written stories they undertook the Relationship Closeness Induction Task (RCIT) [46]. The RCIT is a validated procedure for inducing closeness between strangers under laboratory conditions. Using reciprocal self-disclosure, participants take turns answering a list of questions that become progressively more personal. The RCIT in the current study consisted of 27 questions (e.g., “What is one of your favorite types of food?” leading to more personal topics such as “What is one of your happy childhood memories?”, “What is one accomplishment that you are proud of?”). Answers scripted for the confederate were based on her real experiences but edited to ensure that there was no content relating to sexuality. After completing the RCIT the participant and confederate exchanged and read the second of the written stories.

#### Social distancing measure

After reading the stories, the participant and confederate were asked to take a seat in another room. The confederate always entered first and the participant, seeing the confederate seated, lifted a chair from a stack in the corner of the room. Chalk powder had been placed under the legs of the chair so that its placement could be identified. The physical distance the participant placed between herself and the confederate served as the *social distance* measure [50], with the chairs measured center to center.

#### Post-interaction questionnaire

Once the participant was seated, the experimenter entered and administered questionnaires involving the manipulation check and contact with same-sex attracted men and women (the terms *gay females* and *gay males* were used for participants). Those who had experienced such contact in their daily lives were presented with the additional question “is any of this contact close, such as with a friend or relative?” (*yes* or *no*). Participants were then presented with the question “to which sex are you sexually and/or romantically attracted?” (*opposite-sex*, *same-sex*, *both sexes*, or *unsure*).

## Results and Discussion

Two participants did not pass the manipulation check (conditions: 1- early, 1- delayed) by indicating that the confederate was heterosexual, and one participant (early condition) reported being same-sex attracted. Data from the remaining seventeen heterosexual women (mean age = 18.5 years, *SD* = .87) were retained.

Although only 29.4% of these women reported having no close contact with same-sex attracted males, two-thirds (66.7%) reported having no close contact with same-sex attracted individuals of their own sex. Bivariate correlations for all measures are presented in Table 1.

**Table 1:** Prestudy Bivariate Correlations.

Variable	1	2	3	4	5
1. RWA	−				
2. SIAS	-.32	−			
3. Close contact with gay individuals of own sex (no, yes)	-.03	.28	−		
4.Close contact with gay individuals of the opposite sex (no, yes)	-.15	.43^†^	.48^†^	−	
5. Timing (early vs. delayed)	.03	-.00	.20	-.09	−
6. Social distancing	-.09	.19	.05	.50*	.39

*Note*. *N = 17*. *Female participants interacting with female confederate*. *Higher scores for dichotomous*

*variables = close contact and delayed timing*.

^†^ *p <* .*10*

*p < .*05*

**Fig 1:**
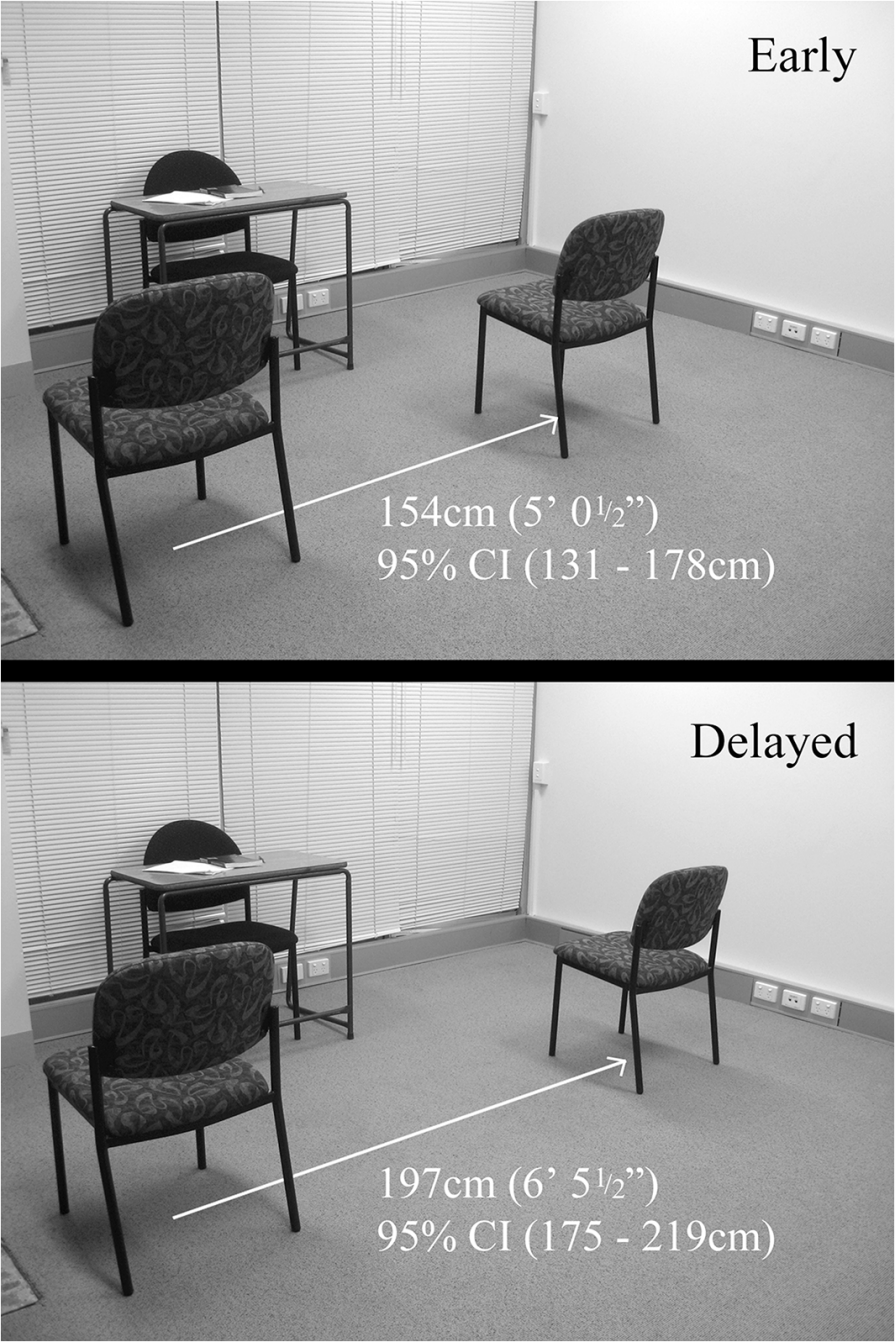
Social distance between participants and confederate according to disclosure timing.

An analysis of covariance (ANCOVA) was conducted to assess the effect of disclosure timing on social distance, while controlling for close contact (i.e. close contact in real life) with same-sex attracted individuals of one’s own sex (i.e. women) and of the opposite sex. There was a trend towards significance for close contact with same-sex attracted women in predicting less social distance, *F* (1, 13) = 3.64, *p* = .079, ƞ^2^ = .10. Close contact with same-sex attracted people of the opposite sex to the participants significantly predicted greater social distance, *F* (1, 13) = 12.22, *p* = .004, ƞ^2^ = .35. In controlling for these covariates, a significant main effect of experimental condition on social distance was observed, *F* (1, 13) = 7.95, *p* = .014, ƞ^2^ = .22 (Fig 1). Results showed that participants sat closer to another female when this female disclosed her sexuality prior to becoming better acquainted (i.e., early) rather than after (i.e., delayed). Further, when also controlling for RWA, *F* (1, 11) = .007, *p* = .933 and SIAS, *F* (1, 11) = .012, *p* = .916, the effect of timing of disclosure on social distancing held, *F* (1, 11) = 6.75, *p* = .025, ƞ^2^ = 22.

## Study 1

In our prestudy, the confederate disclosed a same-sex dating partner during a face-to-face interaction. To ensure the confederate was blind to the experimental conditions, the disclosure occurred through the exchange of a written story in an envelope. In reality, self-disclosure during a social encounter is likely to be conveyed orally. To allow for this, as well as testing of the hypotheses with a larger sample, male and female participants in Study 1 exchanged personal information via computer with a same-gender confederate who disclosed to the participant either early or late during a pre-recorded videoclip. As the stories in which the confederate ‘came out’ in our prestudy were both fairly light-hearted, the *context* of the disclosure was also manipulated, with the story in which disclosure was embedded being either casual or more intimate. As research suggests that intimate information conveyed early between strangers may be considered inappropriate [38], we wanted to test if the current method of embedding disclosure in a relevant topic of conversation would allow early disclosure to be beneficial even when conveyed in a more personal context. Given the larger sample, we used a practical alternative to actual contact. Participants were led to believe they would soon meet the confederate for a face-to-face discussion (i.e., anticipated contact).

### Method

#### Cover story

Participants, who arrived in groups, were advised that they would each be allocated a task partner who had volunteered for course credit to be filmed as part of a ‘get to know you’ study. Participants were told, “In order to prepare for the task, the filmed students were given half the interview questions 30 minutes before filming. The remaining questions were presented during the filming to allow for some spontaneity”. The male and female confederates, being stage actors (although genuinely same-sex attracted), acted in accordance with the above by providing some answers to the list of questions quite readily, pausing for others, and on two occasions stating that they couldn’t think of anything. To ensure the filmed confederates were blind to the timing of disclosure condition, questions answered by the confederates were filmed in segments. The appropriate disclosure segment was then slotted into the film in the correct order. Splicing was disguised through having all questions displayed on the screen for the participant to read prior to a confederate’s response.

As participants were informed that they would later meet the filmed student with whom they were about to be paired via computer, before starting the computer task, the experimenter explained that she needed to first check how many of the filmed students had turned up in a nearby room for the scheduled face-to-face discussion. While appearing to be talking on her cell phone, the experimenter did a quick head count of the participants and said “great, we have (mentions number of participants in the room), thanks Sue (the name of the fictional research assistant), see you soon”. She then told participants “Okay, we’re in luck today. Everyone has turned up at the other end, so you will all be allocated a separate task partner”. Once participants completed the pre-interaction questionnaires, the computer display suggested they were being randomly allocated a task partner from the list of ‘available students’. Discussions during debriefing sessions, which encouraged participants to talk freely about their experiences during the experiment, suggested that the cover story was extremely effective. The small numbers of participants who stated or hinted they had some suspicions were excluded from the analyses (6 women, 3 men).

#### Relationship Closeness Induction Task

Participants were 215 heterosexual undergraduates (90 men, 125 women; mean age = 18.75, *SD* = 1.14). The lab sessions were conducted in same-gender groups of no more than seven. Participants sat in private cubicles and were each randomly allocated to one of the disclosure conditions. After completing the same pre-interaction questionnaires used in the prestudy, participants watched 14 minutes of footage, in which the same-gender confederate read out and answered 21 questions that became progressively more personal (the full list of questions and responses is provided in the Appendix [S1 Appendix]). Participants reciprocated by providing written information about themselves on topics of their choice to present to the confederate. They were led to believe that they would discuss topics of interest during the anticipated meeting. The confederate’s questions and answers remained the same for all conditions. To make the interaction feel more personal, the male and female confederates were instructed to look straight at the camera and say “hi” and answer questions from a list as if sharing the information directly with their task partner (i.e., the participant).

#### Disclosure manipulations

In the pre-recorded footage, the confederate either revealed the gender of a same-sex partner when answering the fourth question (*Early* condition) or the 20^th^ question (*Delayed* condition). As in our prestudy, the sexual orientation disclosure was simply integrated into a broader but relevant topic of conversation. In our first study, however, disclosure took place when discussing a light-hearted experience. To test if the effect of timing of disclosure may vary based on whether the story in which disclosure is embedded is more intimate or more casual, the context of disclosure was also manipulated, resulting in a 2(*Timing*) x 2(*Context*) independent between-groups design.

In the *Intimate* condition, the male or female confederate answered a general question about family members disapproving of any of their dating partners. *“I remember the first time I brought home someone (disclosure—this girl/guy) I was interested in at the time*. *Some of my family seemed cool but I remember it was difficult because I knew that the others really disapproved (disclosure—of her/him)*. *It caused a bit of a clash”* [S1 and S2 Audios]. In the *Casual* condition, the question was about disastrous cooking experiences. In this case, the response was *“I had these friends over for dinner and decided it was my turn to do the cooking*, *so I sent my partner (disclosure–this guy/girl) that I was dating at the time off to buy the food*. *They (disclosure-he/she) came back with this really expensive cut of beef*. *So I made a really tricky beef dish and I was pretty proud of myself but then I found out that two of my guests were vegetarians*!*”* [S3 and S4 Audios]. Table 2 describes the different disclosure conditions.

**Table 2:** Manipulation of Timing and Context for Disclosure of Same-Sex Partner.

Context	Timing of Disclosure	
	Early	Delayed
**Cooking Story**	*n* = 54 confederate mentions gender of partner early (also hears ‘visit home’ story toward end of video but without disclosure)	*n* = 52 confederate mentions gender of partner late (also hears ‘visit home’ story early in video but without disclosure)
**Visit Home Story**	*n* = 55 confederate mentions gender of partner early (also hears ‘cooking’ story toward end of video but without disclosure)	*n* = 54 confederate mentions gender of partner late (also hears ‘cooking’ story early in video but without disclosure)
**Total**	*n* = 109	*n* = 106

#### Dependent measures

To measure *liking* prior to any disclosure of sexuality, after the confederate answered the first of the 21 questions, participants were asked, “How much are you looking forward to meeting this person?” (1 = *not at all* to 7 = *very much*) followed by “What is your current impression of this person?” (1 = *not at all positive* to 7 = *very positive*). These two items served as the *liking* pre-measures. At the end of the video (i.e., after the last question was answered by the confederate), participants were asked, “How much do you *like* this person?” (1 = *not at all* to 7 = *very much*).

As a measure of comfort with the *level of intimacy*, participants indicated their preference in hearing more about the confederate during the anticipated face-to-face discussion on four different topics after viewing the videoclip. Participants were instructed to prioritize by providing their level of interest in hearing more about each of the topics relative to the others (1 = *not interested* to 7 = *very interested*). They were led to believe that this information would be conveyed to their task partner (the confederate) to facilitate the discussion. The topics were determined by pilot research to be either low in intimacy (“where they would like to travel”, “friends”) or high (“one thing they would like to change about the way they were raised”, “qualities to look for in a romantic partner”). Given these topics were presented in the context of sexual orientation disclosure, intimacy in relation to the latter topics was expected to be heightened. As with our prestudy, the manipulation check and contact questions were presented at the end of the experiment.

### Results and Discussion

For all experiments, we also conducted analyses when controlling for participants’ experiences of close contact with both same-sex attracted males and females. *Quality* of contact was also measured (1 = *very negative* to 5 = *very positive*), for those who reported knowing at least one same-sex attracted male or female. Analyses including *quality* measures, however, represent a subset of participants with some level of contact and reduce the sample sizes across experiments by, on average, one-third. Although main effects were found for *quality* of contact measures, these variables did not significantly interact with timing of disclosure.

In keeping with contact theory, among the subset of participants who reported having some level of contact with same-sex attracted individuals of their own sex (*n* = 171), more positive (i.e. *quality*) contact was associated with a greater liking of the confederate, *r =* .38, *p* < .001. Consistent with research suggesting that intergroup interventions to improve heterosexual attitudes towards the same-sex attracted are more likely to be impactful when contact between these groups involves individuals of the same sex [42, 43], more positive contact with same-sex attracted individuals of a different sex to one’s own (*n* = 166) was not associated with greater liking of the confederate, *r* = .06, *p* = .450. Table 3 provides the bivariate correlations for all measures.

**Table 3:** Study 1 Bivariate Correlations.

Variable	1	2	3	4	5	6	7	8	9	10	11	12
1. Gender	−											
2. RWA	-.06	−										
3. SIAS	-.11	.01	−									
4. Close contact with gays—own sex	.11	-.15*	-.03	−								
5. Close contact with gays—opp sex	.20**	-.10	.02	.21**	−							
6. Quality contact with gays—own sex	.20**	-.26**	-.07	.37***	.22**	−						
7. Quality contact with gays—opp sex	.21**	-.10	-.04	.03	.33***	.47***	−					
8. Like pre-measure A (1^st^ impression)	.23**	-.10	.05	.20**	.12	.26**	-.00	−				
9. Like pre-measure B (looking forward to meet)	.22**	-.10	-.18**	.22**	-.05	.25**	-.01	.45***	−			
10. Timing (early vs. delayed)	.01	.05	.10	-.08	-.04	-.11	-.08	.01	.11	−		
11. Context (casual vs. intimate)	.01	.07	-.02	.02	.04	.12	.13	-.14*	-.05	.00	−	
12. DV—Like (post-measure)	.22**	-.29***	-.04	.24***	.03	.38***	.06	.52***	.66***	-.06	-.13	−
13. DV—Discussion topic preference	.05	-.07	-.09	-.01	.04	-.01	.16*	-.20**	-.18**	-.16*	.15*	-.11

*Note*. *N = 215*. *Quality contact gays—own sex*, *n = 171*, *Quality contact gays—opposite sex*, *n = 166*. *Higher scores for dichotomous variables = female*, *close contact*, *delayed timing*, *and intimate context*. *Discussion topic preference in this instance is the difference between the scores for preference for low intimacy topics and high intimacy topics*, *with lower scores = preference for lower intimacy*

*p < .*05*

**p < .*01*

***p < .*001*

In terms of the whole sample (*n* = 215), consistent with our prestudy, approximately two-thirds (65.1%) of participants reported that they did not have any close contact (e.g., friend or relative) with same-sex attracted individuals of their own sex (male participants = 71.1%, female participants = 60.8%). Preliminary analyses indicated that close contact with same-sex attracted individuals of either sex were not significant control variables for the dependent measures of *liking* (when controlling for liking pre-measures), *F*s < 1.37, *p*s > .244, and *topic preferences*, *F*s < .136, *p*s > .713. As such, they were not included in subsequent analyses.

In the following analyses we include gender, timing and context as between-subjects factors (non-significant findings for gender and context as discussed further on). An ANCOVA, controlling for an initial desire to meet the confederate and earlier impressions (i.e. *liking* pre-measures), revealed a main effect for disclosure timing on liking *F* (1, 205) = 7.05, *p* = .009, ƞ^2^ = .03. Those experiencing early disclosure liked the confederate more (*M* = 4.74, *SE* = .09) than those experiencing delayed disclosure (*M* = 4.42, *SE* = .09).

**Fig 2:**
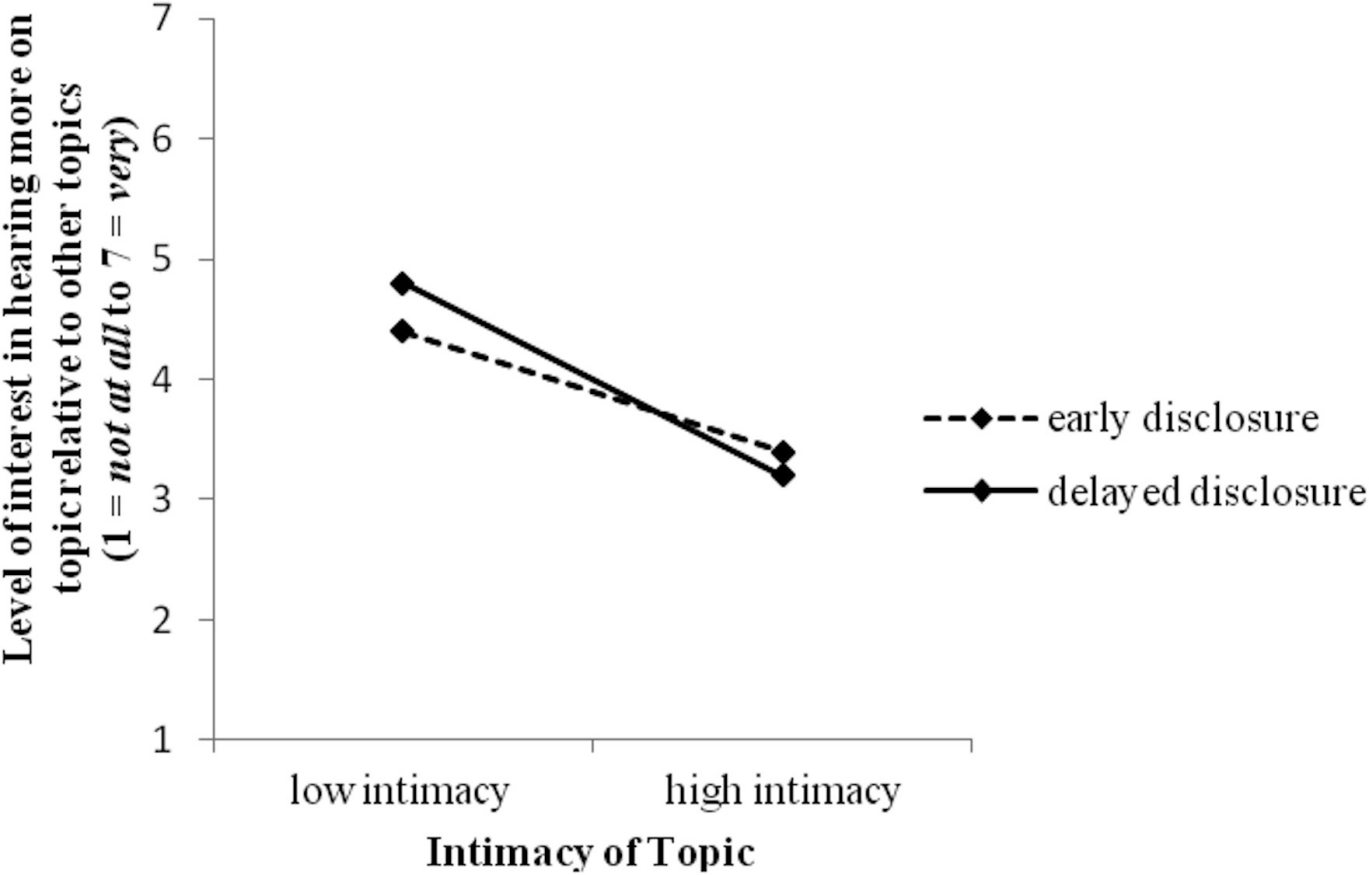
Interaction between disclosure timing and (within-group) topic intimacy level on participants’ preference for discussion topics.

To assess participants’ comfort with level of intimacy, two composite measures—interest in low intimacy topics and high intimacy topics—were created. A repeated-measures ANOVA on interest in low and high intimacy topics revealed a significant interaction between intimacy of topics and disclosure timing, *F* (1, 207) = 6.55, *p* = .011, ƞ^2^ = .03. Those experiencing delayed disclosure indicated a greater preference for hearing about low intimacy topics when meeting the confederate than those experiencing early disclosure. Simple effects analysis revealed that although those experiencing early disclosure also preferred to hear about the low intimacy topics, relative to high intimacy topics, *F* (1, 105) = 47.39, *p* < .001, ƞ^2^ = 31, this effect was stronger for the delayed disclosure group, *F* (1, 102) = 116.76, *p* < .001, ƞ^2^ = 52 (Fig 2).

Study 1’s findings concur with our prestudy. When finding out about the person’s same-sex sexuality earlier rather than later, participants liked the same-gender confederate more and were less likely to want the confederate to prioritize the discussion of relatively superficial topics during the anticipated face-to-face meeting. Further, these results applied to both men and women, with there being no significant interactions between gender and disclosure timing (*F*s < .807, *p*s >.369). As in our prestudy, the results for timing held when RWA and SAIS were controlled for in the analyses, i.e., effect of timing on *liking F* (1, 203) = 7.33, *p* = .007, ƞ^2^ = .03 and effect of timing on *topic preferences F* (1, 205) = 5.71, *p* = .018, ƞ^2^ = .02.

The context of the disclosure did not, however, interact significantly with timing for either of the dependent variables, including any 3-way interactions involving gender (*F*s < 1.63, *p*s > .202), indicating that early disclosure was received more positively, irrespective of whether the topic of conversation was casual (cooking experience) or more personal (bring a dating partner home). Revealing “too much too soon” between strangers may be a violation of social norms [38]. However, the fact that the conversation was relevant (i.e., the confederate was specifically asked about family reactions to dating partners) and the disclosure was simply embedded in the broader topic of discussion, suggest that under these circumstances being upfront about one’s sexuality may be beneficial even when conveyed in a more intimate context.

## Study 2

The results of our prestudy and Study 1 suggest a beneficial effect of early disclosure. One possibility, however, is that in the late condition there is little time to contemplate the disclosure. In both studies, late disclosure participants completed the dependent measures almost immediately after learning of the confederate’s sexuality. Although in reality a person who experiences early disclosure will always know for longer than a person who finds out later, it may be that once an individual is allowed to digest the information his or her initial reactions could change.

In Study 2, we sought to account for this alternative explanation by allowing further time for contemplation. Although both those experiencing early and delayed disclosure become aware of the confederate’s sexuality for an additional length of time (as would be the case in the passing of time in real life), this strategy works to remove the immediacy of the disclosure for those in the delayed condition. Additionally, various dependent measures were included to test the robustness of the beneficial effect of early disclosure. Further, we explored perceptions of the target’s character to examine mechanisms behind heightened positivity to this early timing of disclosure.

### Method

Participants were 221 heterosexual undergraduates (90 men, 131 women; mean age = 18.35, *SD* = 1.13). Study 2’s procedure was identical to Study 1, up to the end of the video session, which included the exchange of personal information and the *liking* measure. To examine the underlying mechanisms of preferred disclosure timing, participants also indicated to what extent they perceived the confederate to be *honest*, *outspoken*, *approachable*, *well-adjusted*, *trusting*, *friendly and high in self-esteem* (1 = *not at all* to 9 = *very*). They were also asked about their *willingness to introduce (the confederate) to friends* on and off campus (1 = *not at all* to 9 = *definitely*). The two ‘introduce to friends’ items were averaged to form a single measure (α = .89), as the two items were strongly positively correlated *r* = .80.

With the aim of providing extra time to contemplate the disclosure, participants were then asked if they would complete a brief task for another researcher. The 12-minute cognitively non-demanding *‘scribble’ task* comprised of connecting random dots and free-hand doodling. Participants then resumed the main study.

#### Dependent measures (after delay)

To assess feelings regarding the anticipated face-to-face discussion, participants then completed Bradley and Lang’s [51] Self-Assessment Manikin (SAM) as non-verbal measures of valence and arousal. The SAM uses graphic figures at various points along 9-point scales, ranging from smiling to frowning for valence, and from a very small to a very large jagged shape within the center of the figure for arousal. As arousal can relate to either positive or negative emotions, the interpretation of the findings is based on the results for valence. Finally, as a *proxy for social distance*, participants were asked to place a card in an envelope indicating whether they would like a one-on-one or a group-of-four discussion (participants of the same gender) when meeting the confederate. Participants were informed that we were interested in conducting discussions under both conditions (i.e., to reduce potential social desirability). At the end of the experiment, participants were presented with the same manipulation check and contact questions used in the two previous studies.

### Results and Discussion

Preliminary analyses indicated that as in Study 2 the context of the disclosure did not significantly interact with timing for any of the dependent variables. Therefore, the results for timing are collapsed for the context variable (early, *n* = 111; delayed, *n* = 110).

Consistent with contact theory, among the subset of participants who had some level of contact with same-sex attracted individuals of their own sex, more positive contact (i.e. covariate *quality of contact*) was associated with more positive scores on several of the dependent measures: liking, *r* = .36, *p* < .001; willing to introduce confederate to friends, *r* = .31, *p* < .001; and feeling happy about meeting the confederate, *r* = .27, *p* < .001. More positive contact with same-sex attracted individuals of a different sex to oneself was associated only with willingness to introduce the confederate to friends, *r* = .16, *p* = .027. Table 4 provides the bivariate correlations between all measures.

**Table 4:** Study 2 Bivariate Correlations.

Variable	1	2	3	4	5	6	7	8	9	10	11	12	13	14
1. Gender	−													
2. RWA	-.02	−												
3. SIAS	.13*	.01	−											
4. Close contact with gays–own sex	.01	-.08	-.03	−										
5. Close contact with gays–opp sex	.17*	-.12	-.01	.23***	−									
6. Quality contact with gays–own sex	.18*	-.17*	-.12	.47***	.17*	−								
7. Quality contact with gays–opp sex	.10	-.09	-.15*	.14	.33***	.42***	−							
8. Like pre-measure A	.23**	-.03	.03	-.06	.04	.16*	.01	−						
9. Like pre-measure B	.21**	-.14*	.03	.04	.07	.37***	.11	.58***	−					
10. Timing (early vs. delayed)	.02	-.04	.05	-.03	.11	-.04	.09	-.04	.08	−				
11. DV—Like (post-measure)	.22**	-.17*	-.00	.00	.04	.36***	.09	.65***	.77***	-.08	−			
12. DV—Introduce to friends	.24***	-.18**	.02	.10	.13	.31***	.16*	.49***	.51***	-.12	.59***	−		
13. DV—Happy to meet	.17*	-.00	-.19**	-.02	.06	.27***	.14	.61***	.59***	-.16*	.61***	.58***	−	
14. DV—Excited to meet	.22**	-.09	.20**	.01	.12	.16*	.02	.40***	.43***	-.13	.45***	.42***	.36***	−
15. DV—Prefer meet alone vs. in group	.05	.03	.16*	.04	-.07	-.14	-.05	-.20*	-.18*	.17*	-.20**	-.10	-.25**	-.07

*Note*. *N = 221*. *Quality of contact with gays—own sex*, *n = 177*, *Quality of contact with gays—opposite sex*, *n = 181*. *Higher scores for dichotomous variables = female*, *close contact*, *delayed timing*, *and meet confederate in group (rather than alone)*.

*p < .*05*

**p < .*01*

***p < .*001*

In terms of the whole sample, as with the previous two studies, close to two-thirds of participants reported that they did not have any close contact (e.g., friend or relative) with a same-sex attracted person of their own sex (male participants = 63.3%, female participants = 62.6%). Further, as in Study 1, close contact with same-sex attracted individuals of either sex were not significant control variables and results held when controlling for RWA and SIAS (see SAM results for analysis retaining SIAS).

Consistent with Study 1, when controlling for an initial desire to meet the confederate and earlier impressions (i.e. *liking* pre-measures), the main effect for timing on *liking* (measured before the 12-minute waiting period) was replicated, *F* (1, 215) = 10.21, *p* = .002, ƞ^2^ = .04, with recipients of early disclosure liking the confederate more (*M* = 5.85, *SE* = .10) than those experiencing delayed disclosure (*M* = 5.40, *SE* = .10). There was no significant interaction effect of timing with gender, *F* (1, 215) = 2.19, *p* = .140.

To explore the mechanisms behind the preference for early disclosure, a multivariate analysis of variance (MANOVA) was conducted on participants’ perceptions of the confederate’s character. Results revealed no effect of disclosure timing on any of the characteristics (*honest*, *outspoken*, *approachable*, *friendly*, *well-adjusted*, *trusting or high self-esteem)*, with all *F*s < 2.14 and all *p*s >.145. Further, there were no significant gender differences, with all *F*s < 4.97 and all *p*s > .089. It is interesting to note, however, that apart from *self-esteem*, all trait scores, although not significantly different according to timing, were in the expected direction, with lower scores for delayed disclosure. Given this, a composite measure of the traits was created (α = .82). Consistent with the MANOVA findings, an ANOVA found no significant effect of timing of disclosure, *F* (1, 217) = .986, *p* = .332, or an interaction effect of timing with gender, *F* (1, 217) = .010, *p* = .921, on the global evaluation score.

To assess participants’ feelings just prior to the anticipated meeting with the confederate (i.e., after the 12-minute waiting period), a multivariate analysis of covariance (MANCOVA) was conducted on the SAM measures of valance and arousal, while controlling for general social interaction anxiety (SIAS), Wilks’ Lambda = .88, *F* (2, 215) = 14.31, *p* < .001. Significant multivariate effects for timing, Wilks’ Lamda = .97, *F* (2, 215) = 3.13, *p* = .046, were followed up with univariate analyses. Those who experienced early disclosure were happier (*M* = 6.01, *SE* = .14), *F* (1, 216) = 4.22, *p* = .041, ƞ^2^ = .02 and more excited about the meeting (*M* = 4.59, *SE* = .16), *F* (1, 216) = 4.38, *p* = .037, ƞ^2^ = .02 than those who experienced delayed disclosure (Happy, *M* = 5.66, *SE* = .14; Excited, *M* = 4.11, *SE* = .16). Multivariate effects for gender x timing were not significant, Wilks’ Lamba = .99, *F* (2, 215) = .54, *p* = .542.

**Fig 3:**
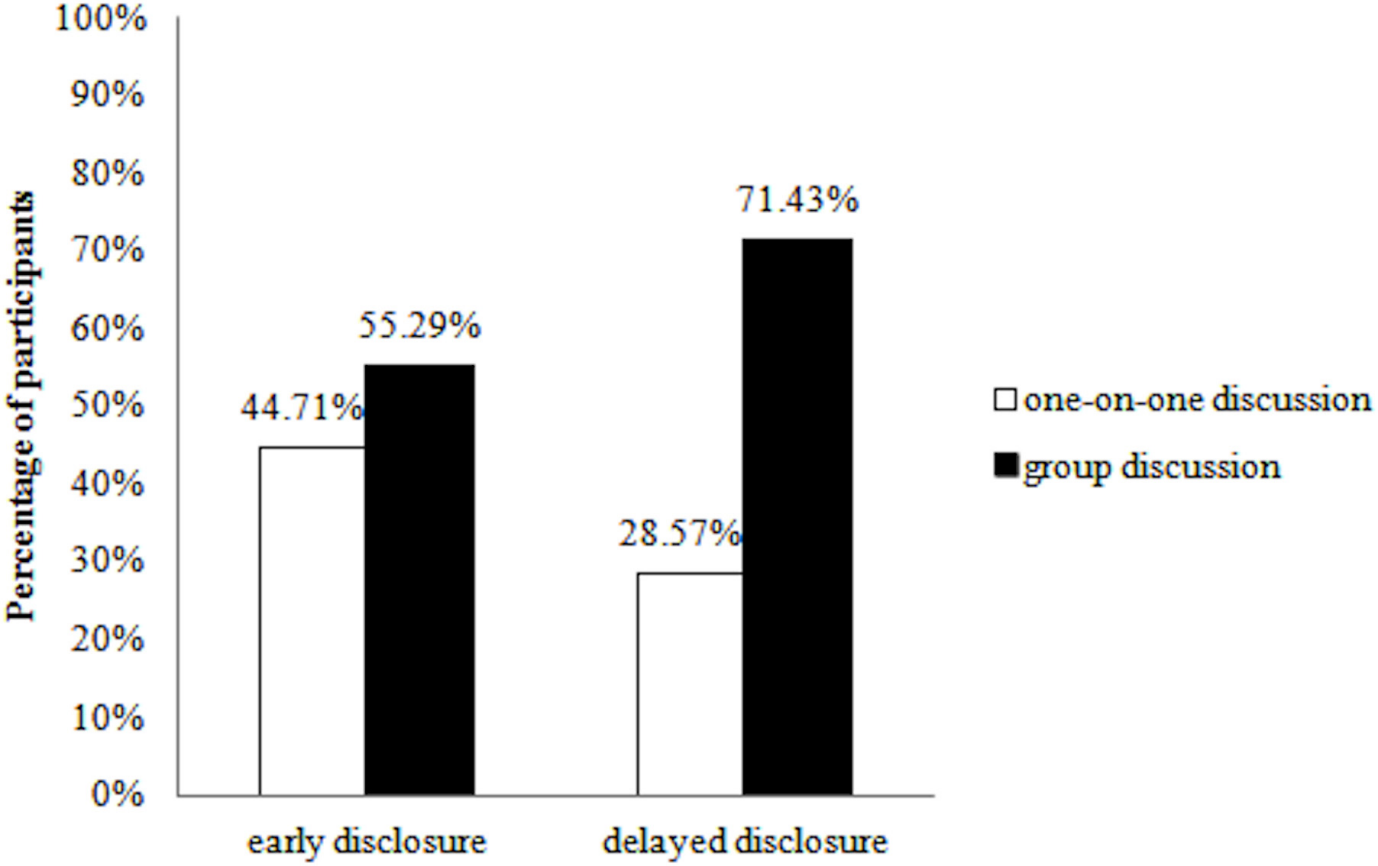
The effect of disclosure timing on preference for one-on-one or group meeting with the confederate.

A loglinear analysis of participants’ preferences for meeting the confederate alone or in a group (proxy for social distance) was conducted, using backward elimination (i.e., beginning with all effects and then the removal of higher-order interactions, such as a 3-way, to assess if its removal has a significant effect on the fit of the model. The analysis stops once a higher-order interaction is found, as with categorical data all the lower-order interactions are consumed within those of a higher order [52]). The final model had good fit between the expected and observed frequencies, χ^2^ (4) = .677, *p* = .954. Backward elimination revealed a significant association between disclosure timing and preferred group size χ^2^ (1) = 4.77, *p* = .029 (with the higher-order interaction of gender x timing x preferred group size, not significant, χ^2^ (1) = .010, *p* = .921). Odds ratios indicated that those who experienced early disclosure were twice as likely (2.02) to select a one-on-one discussion than those who experienced delayed disclosure (Fig 3).

A significant interaction between gender and timing was found only on *willingness to introduce to friends*, *F* (1, 217) = 5.02, *p* = .026, ƞ^2^ = .02. Women were significantly more willing to introduce the confederate to their friends when disclosure occurred earlier (*M* = 5.49, *SD* = 1.70) rather than later (*M* = 4.60, *SD = 1*.*51*), *p* = .004, ƞ^2^ = .07. There was no significant effect of timing on this measure for men, (Early, *M* = 4.05, *SD = 1*.*99*; Delayed, *M* = 4.25, *SD* = 1.95), *p* = .599, ƞ^2^ = .00.

## Study 3

Results from the prestudy and Studies 1 and 2 revealed a benefit for upfront disclosure of a person’s same-sex sexual orientation. The mechanism that underpins more positive reactions to this early timing however, remained unclear. In our attempt in Study 2 to identify such a mechanism, a deliberative (i.e., explicit) differential self-report measure was used. Although scores were in the expected direction (i.e., lower scores on positive traits relating to the confederate for the delayed condition), differences between the experimental conditions were not statistically significant. Past research has suggested that in studies of socially sensitive issues, such as homosexuality, participants may offer socially desirable responses [53]. Interestingly, and consistent with MacInnis and Hodson [21] scores on RWA–our proxy for prejudice–did not impact the effect of disclosure timing on participants’ reactions. As such, if people are unaware of using stereotypes then these stereotypes will bias the judgements of low-prejudice and high-prejudice individuals alike [54]. Therefore, in this exploratory study we use a very different strategy from the last three experiments, in that we try to identify the underlying reasons for the more favorable experiences of upfront disclosure by employing a more implicit approach.

A less direct means of identifying the reasons behind people’s reactions may be through examining participants’ memories in order to understand their person perception process [55]. In our studies, those experiencing early disclosure learnt everything about their task partner (e.g., friendships, childhood memories, life’s funny experiences) with the full knowledge that he or she was same-sex attracted. Conversely, those who experienced delayed disclosure were left to make sense of the connection after the fact. That is, they had to adjust their initial perceptions of their task partner in light of more recent information.

According to research examining the link between judgement and recall during impression formation [56, 57], those experiencing early disclosure would have formed their impressions using *online impression formation*. That is, they had the opportunity to organise the incoming information using a relevant category at the time the information was being gathered. This form of processing is believed to result in a more global assessment of the individual, rather than one based on the recall of any specific event [58, 59]. On the other hand, participants experiencing delayed disclosure may have tried to recall information about the confederate in order to integrate that information with the newly acquired (‘gay’) category, so that a coherent impression could be formed. In this case, participants would have needed to switch to *memory-based impression formation*. Judgements reliant on memory tend to be based on more salient or attention-getting events, as these are more accessible at the time of judgement [60]. In the context of our studies, gaining information about the confederate’s sexual orientation is likely to be such a salient event. As noted by Herek [39], sexual orientation represents a master status [61], in that once a person is known to be same-sex attracted, it can be regarded by others as one of the most important pieces of information that they have about that individual.

Further, research on *impression formation* and *inconsistency resolution* suggests that those in the delayed condition may have ultimately experienced a more taxing task (i.e., relying on memory to integrate the information), due to a potential mismatch between their initial beliefs (i.e., the person was heterosexual) and those resulting after the disclosure. Several studies have found that when memory relies on ‘free recall’ under cognitive load, stereotype consistent information is recalled better than more individuating information [62–64]. Additionally, when event memories start to fade, people may unconsciously use schemas to complete or embellish these faded memories [65].

Collectively, this research suggests that those who experienced delayed disclosure of the confederate’s sexuality would be very attentive to the fact that the confederate was same-sex attracted. However, they would need to rely on memory to integrate this new information, with this greater mental effort rendering them more likely to recall details that “fit” their ‘gay’ schemas. In contrast, those who experienced early disclosure, and could therefore process all the personal information the confederate shared with them in this light, would be more able to integrate individuating information with the ‘gay’ category and perhaps as a result challenge stereotypes. The information shared by the confederate in our studies was purposefully designed to not include any stereotypical (or atypical) content, so as not to confound the timing of disclosure (i.e., lead to the assumption that the confederate was same-sex attracted prior to the late disclosure event). Therefore, we hypothesise that when instructed to recall the information shared by the confederate, those in the early condition, relative to delayed, will 1) correctly remember more topics of conversation shared by the confederate and 2) will be less likely to incorrectly recall stereotypical information in relation to a same-sex sexual orientation. Although both timing of disclosure groups will be aware of the confederate’s sexual orientation (as revealed by the manipulation checks and feedback during debriefing sessions for all three previous experiments), we expect, in keeping with *memory-based impression formation*, that the confederate’s sexuality would be more salient for the delayed disclosure group, relative to the early, and as a result 3) they would have greater recall of the two stories involving a dating partner, even though one of these stories occurs early in the videoclip without the disclosure of the partner’s gender (i.e. delayed condition).

### Method

The purpose of this fourth experiment was to identify the impact of timing of disclosure on participants’ memory of the information shared by the confederate in the videoclip. As men and women responded similarly to timing of disclosure in our earlier studies, the current study involved only female participants (viewing a female confederate).

Forty undergraduate women (mean age = 18.60, *SD* = 1.39) were randomly assigned to each of the early and delayed disclosure conditions used in Studies 2 and 3 (see Table 2). Prior to the videoclip, participants were presented with the same RWA and SIAS measures.

#### Dependent measures

Following the videoclip, participants were instructed to type as much information as they could recall about what the confederate shared with them (from the 21 topics discussed). As participants may have chosen to only mention the topics they felt confident in remembering, this was followed by questions regarding what they could recall about the two potential disclosure stories involving a dating partner, i.e., the disastrous cooking experience and the bringing a partner home to meet the family experience (e.g., “what happened when the person brought a dating partner home to the family?”). The confederate ‘came out’ in either one of these stories, be it early or delayed. The order in which the questions were presented was counterbalanced.

On completion of this, participants were presented with questions on their overall perception of the accuracy of their recall of the information (“How easy was it for you to recall?”, “How quickly were you able to recall?”, “How confident are you in the accuracy of your recall?”). The response options ranged from 1 = *not at all* to 7 = *very* (α = .83). Participants were then presented with the same concluding questions used in the earlier experiments (i.e., manipulation check, contact with same-sex attracted people, and their own sexual orientation).

### Results and Discussion

Five participants reported having same-sex attractions (2 same-sex only and 3 bi-sexual). Data from the remaining 35 heterosexual women (mean age = 18.57, *SD* = 1.42) were retained for analysis (*n* = 19 early disclosure, *n* = 16 delayed disclosure). All participants passed the manipulation check, selecting the confederate’s sexual orientation as ‘gay’.

Results showed that there were no differences between the early (*M* = 27.13, *SD* = 8.27) and delayed disclosure groups (*M* = 29.04, *SD* = 7.84) in terms of the volume of information recalled, as measured by the number of typed lines, *t* (33) = -.70, *p* = .492.

#### Information correctly recalled

Results also revealed that there were no significant differences between early (*M* = 9.87, *SD* = 3.70) and delayed disclosure (*M* = 10.53, *SD* = 2.77) on the number of topics correctly recalled, with both groups providing correct details for approximately half of the topics discussed by the confederate, *t* (33) = -.59, *p* = .559. With the exception of the stories in which disclosure took place (discussed below), none of the participants provided information on a topic incorrectly. That is, it was either correctly recalled or not recalled at all. Therefore, our first hypotheses that people experiencing early disclosure would correctly recall more information shared by the confederate was not supported.

#### Information incorrectly or not recalled

The two disclosure stories (i.e., the disastrous cooking experience and the visit home to family) were the only two in which the confederate made reference to a dating partner, with gender either not mentioned or disclosed depending on timing. It was for these two stories (with all participants hearing both) that differences were found between the groups in terms of their memory.

*Stereotyping (evidence of embellishment)*. In applying stereotypes, knowledge of someone’s homosexuality will often color other information about that individual, even information totally unrelated to their sexual orientation [39]. In the story about bringing a dating partner home, the confederate simply mentioned that some family members were pretty cool but that some disapproved of a dating partner, causing “a bit of a clash”, with no mention of the reason being due to sexual orientation (for exact wording see Study 2).

All participants correctly remembered that there were negative reactions (i.e., disapproval) from family members. Some participants however, exaggerated the severity of the negative outcome and made inferences based on information that was not provided. This form of embellishment (which was the only exaggerations or fictitious addition to the outcome observed in the data) was coded as 1 and *no embellishment* coded as 0, and two independent coders undertook this rating. An interrater reliability analysis using Cohen’s kappa coefficient was performed to determine consistency between two raters, with Kappa = 0.93, *p* < .001. Based on guidelines for the interpretation of kappa [66], values between 0.81 and 0.99 represent “almost perfect agreement” between raters. Results revealed that no one (0%) in the early disclosure condition embellished on the outcome, whereas 56.3% (*n* = 9) of those experiencing delayed disclosure did. This difference between groups was statistically significant, Fishers exact test (*n* = 35), *p* < .001, Cramer’s *V* = .64. For example, in relation to bringing a dating partner home to family (in which the confederate mentioned “it caused a bit of a clash”), different participants in the delayed group recalled that “it caused a massive clash”, “they were shocked as they didn’t want their daughter/sister to be dating this particular person–she was offended”, “caused a commotion–lots of people disapproved”, “they fought–she felt horrible”, “caused a very big clash”, “the family and the person’s girlfriend didn’t get along”, “the one’s who did not approve of her sexual orientation did not like her partner”.

Embellishment occurred irrespective of whether these participants experienced the delayed disclosure through the ‘visit home’ story or the ‘cooking’ story, Fishers exact test (*n* = 16), *p* = .315. That is, even in cases when these delayed disclosure participants learned of the person’s sexual orientation via the light-hearted cooking story, they were just as likely to negatively embellish the outcome of the ‘visit home’ story. While demonstrating that these results are not simply the effect of the recency of hearing a particular story, these findings are in keeping with our expectation that those in the delayed condition would rely on *memory-based impression formation* (i.e., having to piece together the information shared by the confederate with the newly acquired ‘gay’ category), with this more cognitively demanding task resulting in a greater tendency for people to fall back on their ‘gay’ schemas. While acknowledging the genuine plight of those who experience rejection due to their sexuality, researchers have noted a strong tendency to focus on the negative experiences of being same-sex attracted, with relatively little attention given to positive experiences [67, 68]. Given this attention bias towards negative life outcomes for people who do not identify as heterosexual, such views are likely to dominate, despite a recent U.S. survey showing that the majority of same-sex attracted people reported either stronger or unaffected family relationships since ‘coming out’ [69].

In contrast, those in the early disclosure condition who made reference to the clash among family members either simply mentioned “a clash” occurred (*n* = 4) or correctly minimized the clash (*n* = 5), i.e., “a slight rift”, “a bit of tension”, “some segregation”, “some clashes”, “a bit of a clash”. Therefore, even when those in the early disclosure condition recalled tension between family members this was never exaggerated or associated with the confederate’s sexual orientation. In fact, it was mostly in keeping with the confederate’s statement that it caused “a bit of a clash”.

Interestingly, findings showed that those who embellished the outcome (all belonging to the delayed disclosure group) believed themselves to be more accurate in their recall of the information shared by the confederate (*M* = 5.85, *SD* = .58), compared with those who didn’t embellish (*M* = 5.26, *SD* = .84), *t* (33) -1.96, *p* = .034 [95% CI, 1.12– .07], *d* = .70.

No cases of embellishment were found for the cooking experience scenario for either timing of disclosure group. This was to be expected, however, given the issue of sexuality was not likely to be related to a story about cooking a meat dish for vegetarians.

*Initial recall of disclosure scenarios*. The above findings on the recall of the potential disclosure scenarios were the result of participants being asked what they could remember about these two stories. However, we also wanted to see if people would recall these stories when not specifically asked (i.e., in their initial overall recall of the information shared by the confederate). The majority of those in the early and delayed conditions (84.2% and 87.5% respectively) mentioned the scenario in which the disclosure took place for them, with no difference between groups, Fisher’s exact test (*n* = 35), *p* = 1.00. Differences were found however, when it came to their recall of the scenario in which disclosure had not taken place for them. That is, the story in which a dating partner was mentioned but the partner’s gender was not (be this either in the ‘cooking’ or ‘visit home’ story). In this case, 100% of those who had experienced delayed disclosure remembered this scenario, compared with 57.9% of those in the early disclosure condition, with this difference statistically significant, Fisher’s exact test (*n* = 35), *p* = .004, Cramer’s *V* = .50. For delayed disclosure participants this would have involved recalling the earlier-placed dating partner story (4^th^ topic of discussion–gender not mentioned), where as for early disclosure participants this would have involved recalling the more recently placed dating partner story (20^th^ topic of discussion–gender not mentioned). In other words, despite this information being more distant for those who experienced delayed disclosure, they were more likely to mention the event, relative to those who had experienced the early timing.

This suggests that the non-disclosure dating partner event for participants in this study was more salient for those who experienced delayed disclosure, compared with those who experienced early disclosure. In keeping with *memory-based impression formation*, these participants, having only recently found out that the person was same-sex attracted, perhaps needed to reflect on the earlier dating partner story in order to re-evaluate (i.e., the dating partner was likely to be a woman not a man). In contrast, early disclosure participants would have already known that the person was same-sex attracted by the time the second dating partner story was told. Therefore, no re-evaluation would have been required, rendering it less salient. Instead, and in keeping with *online impression formation*, these participants would have had the opportunity to organise this incoming information using the relevant ‘gay’ category and thereby simply integrate this information into their overall knowledge of the confederate.

Importantly, the above effect was found irrespective of the story in which the participant experienced the gender of a partner not being mentioned (i.e., ‘cooking’ or ‘visit home’ story). As 100% of those in the delayed condition freely recalled the non-disclosure dating partner scenario, this applied to both stories (*n* = 8 and *n* = 8). Although those who had experienced early disclosure were less likely to remember this event, among those who did remember there was likewise no difference between the type of story, with 66.7% recalling the event for which the ‘visit home’ story was relevant and 50.0% recalling the event for which the ‘cooking’ story was relevant, Fisher’s exact tests (*n* = 19), *p* = .650. These findings again show that it was the effect of timing of disclosure on people’s memory of events and not the type of story in which the disclosure was embedded. This is also consistent with our earlier experiments in which we found that it was timing and not the context of the disclosure that had an impact on the dependent measures.

As with our three earlier experiments, measures of RWA, SIAS and close contact with same-sex attracted people of either sex did not contribute to the significant findings: for embellishment (RWA, *p* = .403, and all other *p*s > .680); and the non-disclosure story (RWA, *p* = .133, and all other *p*s > .681). Second, there were no significant differences between the four experimental groups on any of these measures (RWA, *p* = 821, chi-square test for close contact with same-sex attracted person of one’s own sex, *p* = .535, all other *p*s >. 239). This was also the case when comparing just the early vs. delayed timing groups (RWA, *p* = .407, chi-square test for close contact same-sex attracted person of one’s own sex, *p* = .505, all other *ps* > .09).

## General Discussion

As the role of cross-group friendships in promoting positive outgroup attitudes is now well-established [3, 8], including in the case of sexual minorities [17–19, 70] the current studies aimed to identify strategies to help increase the chances of these friendships developing in the first place.

Across three experiments, heterosexual individuals experiencing contact or anticipated contact with a same-sex attracted person of their own sex reacted more positively when the disclosure of the person’s sexual orientation occurred before getting to know them rather than after. Further, the benefits of knowing sooner, rather than later, continued to apply even when participants were given further time to process the disclosure, thereby removing the immediacy of the revelation for those in the delayed group.

The confederate, when disclosing early rather than later, was approached more closely (Prestudy) and liked more (Studies 1–2). Those experiencing early disclosure, compared with later, were less drawn to topics of lower intimacy (Study 1), were happier and more excited about meeting the confederate, and more likely to choose to be alone with the confederate for a one-on-one discussion (Study 2). Further, women experiencing early disclosure, compared with late, were more willing to introduce the same-gender confederate to their friends (Study 2). This was the one dependent measure for which we did not find a significant effect of timing of disclosure for men. Male participants in general, however, were less likely than females to report that they would be willing to introduce the confederate to friends. This may be attributed to the fact that women generally score higher than men on measures of expressive/communal traits (e.g., caring, warm in relations to others) [71].

The overall positive findings for upfront disclosure suggests that early revelation of a same-sex sexual orientation, under the current conditions, is more likely to offer opportunities for continued contact, which can lead to the development of cross-group friendships. Additionally, the generally uniform findings for men and women in our studies adds further weight to the argument that researchers investigating attitudes of behavior in relation to same-sex sexuality need to control for both sex of respondent and sex of target (or attitude object) to provide meaningful results [72, 73].

In our fourth experiment we endeavoured to identify reasons behind the more positive reactions to early disclosure through *person memory*, which examines how memory (recall) can be used to better understand the person perception process [55]. We found no differences between the early and delayed groups in terms of their correct recall of the number of topics shared by the confederate (with both groups remembering approximately half) or their recall of the story in which the disclosure took place. Those in the delayed condition, however, were more likely to negatively embellish information involving a dating partner and have greater recall of the story in which the disclosure had not taken place, relative to those in the early condition. This was despite the non-disclosure story being a more distant memory for the delayed disclosure group, suggesting that perhaps given their recent awareness of the confederate’s sexual orientation, they needed to reflect on the earlier dating partner story in order to re-evaluate in terms of the partner’s gender.

These exploratory findings suggest that although both early and late disclosure participants were very much aware of the confederate’s sexuality, this aspect of the confederate was more salient for the participants in this study who experienced delayed disclosure. These findings are in keeping with our expectation that those in the delayed condition would rely on *memory-based impression formation* (i.e., having to piece together the earlier information shared by the confederate with the newly acquired ‘gay’ category), with this more cognitively demanding task resulting in a greater tendency for people to fall back on their ‘gay’ schemas.

Interestingly, people who negatively embellished information involving a dating partner (all from the delayed disclosure group) believed that their recollection of information shared by the confederate was more accurate, relative to those who didn’t embellish. This finding is also consistent with memory research, which shows that despite memories being false, people can still feel confident in their recollections [74]. Further, and as noted earlier, if people are unaware of using stereotypes (i.e., if they believe the information to be true) then these stereotypes can bias the judgements of low-prejudice individuals just as powerfully as they can for high-prejudice individuals [54].

In contrast, no one who experienced early disclosure embellished information involving the dating partner and they were much less likely to recall the non-disclosure story. In the latter case, and consistent with the consequences of early disclosure in real life, participants would have already known that the person was same-sex attracted by the time the second dating partner story was told, rendering it less salient. In keeping with *online impression formation*, these participants would have had the opportunity to organise this incoming information using the relevant ‘gay’ category and thereby simply integrate this information into their overall knowledge of the confederate.

Although further research is required to test these exploratory findings, they do raise the possibility that the more positive reactions to early disclosure in our first three experiments may have been due to a reduced tendency to focus on the confederate’s sexuality as a defining feature. Although contact theory research emphasises that outgroup salience, at least at some level, is required for positive contact with outgroup members to generalise to the minority group as a whole [75], researchers have more recently focused on an integrated approach. That is, contact should allow for the de-emphasising of group differences through promoting interpersonal closeness, while at the same time allowing for the awareness and appreciation of people’s unique group memberships [9, 28, 41]. In all our experiments, those who experienced early disclosure learnt everything about their task partner (e.g., friendships, childhood memories, life’s funny experiences) with the full knowledge that he or she was same-sex attracted.

Our findings for the benefits of early disclosure are consistent with the experimental studies by Gross et al. [20] and MacInnis and Hodson [21]. However, neither of these studies involved direct disclosure by the same-sex attracted individual nor was the impact of timing tested under conditions of contact. Although MacInnis and Hodson [21] commendably employed a task to induce interpersonal closeness during an online interaction, at no stage were participants given the impression they would ever meet the alleged same-sex attracted individual. Given research has found that anticipating face-to-face contact with a stigmatised other can lead to anxiety-related behavior [36], our current results suggest that upfront disclosure can also be beneficial under these more testing conditions, with in-person contact undeniably important to the development of cross-group friendships.

Our studies also found that the benefits of early disclosure applied to both those with and without close contact with same-sex attracted people (of either gender). As lack of close contact with outgroup members is associated with higher levels of prejudice, this is consistent with findings from additional analyses which revealed that those who scored lower, as well as higher, on RWA (as a proxy for homosexual prejudice) reacted more favourably to early disclosure. This was also found to be the case by MacInnis and Hodson with their sample of undergraduates living in Canada [21]. Although one may argue that RWA was not an appropriate measure of homosexual prejudice for our young sample of Australian students, bivariate correlations suggest this wasn’t this case. For example, overall, those scoring higher on RWA liked the same-sex attracted confederate less (Studies 1 and 2) and were less likely to want to introduce this person to their friends. They were also more likely to report experiencing negative contact with same-sex attracted people of their own gender (covariate–*quality* of contact). This is consistent with other student sample studies in Australia and nearby New Zealand which found higher scores on authoritarianism predicted higher levels of homosexual prejudice [76, 77].

Our results, however, are inconsistent with earlier studies that found benefits for delayed disclosure [22–24]. In our analyses of these papers we argued that the methodologies used in these previous studies did not allow for the benefits of early disclosure to be fully captured. That is, these studies did not involve a confederate personally sharing information about themselves and their life experiences with a participant. Not only do people tend to feel greater liking towards others who disclose to them [37], a meta-analytic review on intergroup contact research suggests it is key to the development of cross-group friendships and thereby the improvement of outgroup attitudes [8]. Further, in these previous studies the person’s sexuality was a focal point of the information shared. This less personal form of interaction and more explicit disclosure, in defying social norms [38], may have resulted in a more negative perception of the same-sex attracted person from the very start and tainted all forthcoming information (i.e., a primacy effect). In contrast, the disclosure in the current studies may have been in a sense legitimized through being naturally integrated into a broader topic of conversation, in others words, embedded in the detail of everyday life.

The findings from the current set of experiments paint a fairly positive picture for the early revelation of a person’s same-sex attractions, at least in terms of low-risk social encounters conducive to extending one’s friendship networks. However, they do not constitute advice on how or when to disclose a same-sex sexual orientation. First, our studies examined the impact of the timing of such disclosure solely from a heterosexual individual’s perspective. Studies show that the experience of intergroup contact for dominant group members is not necessarily the same as it is for those belonging to a stigmatised minority [4, 7]. Further, the disclosure in our studies involved interaction with a new contact and a situation in which the same-sex attracted person did not have a vested interested in the relationship. Disclosure during a job interview, for example, may pose greater risk. What these studies do suggest is that there may be some merit in the fact that more and more same-sex attracted people are making their sexual orientation known [78], and in much the same way as heterosexual individuals convey the nature of their relationships in their everyday interactions with others [40]. With the number of countries and jurisdictions recognising same-sex marriages increasing rapidly [79], disclosing the gender of one’s spouse as a natural part of a broader topic of everyday conversation, is likely to become even more commonplace.

It is important to acknowledge that, although our findings were statistically significant, the effect sizes for timing of disclosure in several cases were small (e.g., in Studies 1 and 2, timing in many cases explained 2–4% of variance in our dependent variables). However, for our dependent measures that related to having contact with the same-sex attracted confederate, the effect sizes were larger. For example, when we measured how far away a participant sat from the confederate (in our prestudy), timing of disclosure explained 22% of the variance in distance. When it came to introducing the confederate to friends, among female participants, timing of disclosure explained 7% of the variance in ‘willingness to do so’. Further, when participants were asked to select whether they wanted to be alone or in a group for their anticipated meeting with the confederate, odds ratio results showed that male and female participants who experienced delayed disclosure were twice as likely to not want to meet the confederate alone. This suggest that while timing of disclosure may have a more subtle impact on some measures, such as, how much one liked the confederate or preferred topics of conversation with the confederate, timing of disclosure is likely to have meaningful impact in situations that involve heterosexual contact with a same-sex attracted individual.

As with all laboratory studies, however, these experiments may have some limitations in terms of their generalizability to the real world. When an individual meets someone for the first time, the opportunity, or will, to continue interacting with the person may not always be present. Therefore, should people react somewhat negatively to early disclosure at the start there may not always be the opportunity to rectify this first impression through getting to know a little more about that individual. However, the risks during social encounters are likely to be greater for delayed disclosure going awry than early. For example, if early disclosure is not well received, the person disclosing has lost little other than the potential to start a new friendship. In the case of delayed disclosure, this can result in the same-sex attracted individual losing an existing and highly valued relationship [80, 81].

Our findings also suggest that early disclosure may be beneficial when embedded in a fairly intimate as well as non-intimate context. In reality, intimate discussions are less likely to occur when getting to know someone for the first time. Nonetheless, the findings from this research suggest that provided the information is embedded in a relevant and broader topic of discussion, early disclosure during more intimate conversations may still be beneficial. More importantly, however, our research shows that early disclosure is likely to be received more positively than delayed, under circumstances that are very much in keeping with how same-sex attracted and heterosexual people alike may choose to casually convey who they are in terms of their relationships with others when meeting people for the first time.

It is important to acknowledge, however, that the confederates in our studies were depicted as neither typical nor atypical of their group membership, in order to test for the timing of disclosure of a concealable stigma. If a same-sex attracted individual is considered highly typical of their group on first appearance (e.g. mannerisms, clothing), an interactant may strongly suspect, or even assume, the person’s outgroup status. It may be that timing of disclosure has little effect in this case, given the heterosexual individual may feel they already know. Alternatively, although our studies did not find that timing impacted participants’ perceptions of the confederate’s honesty, it may be that early disclosure under these more conspicuous conditions also receives more favorable reactions, as delaying disclosure may be considered by some heterosexual individuals to be ‘holding back’ (i.e. it’s obvious, so why not mention it?). Therefore, further research is required to examine the impact of timing of disclosure of sexuality on heterosexual individuals’ reactions, when the same-sex attracted person is considered stereotypical of their group.

It is also important to note that the findings from the current research involved young heterosexual individuals, who in general have more positive views regarding same-sex attraction [82]. It would be interesting to test these findings with mature-aged heterosexual individuals experiencing contact with their same-sex attracted counterparts. Further, Australia, as a nation, has fairly positive attitudes toward sexual minorities [82]. It would be beneficial to see if findings differ for nations in which homosexuality is highly stigmatised.

Although our first study (prestudy), involving actual contact with a same-sex attracted confederate, found a significant effect of timing of disclosure that was consistent with the results from our larger ‘anticipated contact’ studies, the sample size was small. Therefore, we recommend that future research replicate the study with a larger sample, using the same approach, to address issues of generalizability. Additionally, our last study was exploratory, revealing what appears to be a greater tendency for those experiencing delayed disclosure to focus on factors related to the confederate’s sexuality. Therefore, we strongly encourage other researches to further test this possibility, by conducting larger scale implicit design experiments on the timing of disclosure of a same-sex partner.

It would also be of value to see if these findings extend to other types of concealable stigmas, or even traditionally non-concealable stigmas, such as racial/ethnic minority status, rendered invisible through contact increasingly initiated via the internet. It is acknowledged, however, that the disclosure of a person’s sexuality can be as simple as using the correct pronoun when referring to a current or past partner. Disclosure of other concealable stigmas, such as mental illness, is likely to be more complex. Nonetheless, given the negative health consequences of having to keep one’s stigmatized status hidden [83, 84], research on the effects of timing of disclosure for other concealable outgroup memberships, under similar social conditions, is of equal importance and worthy of investigation.

## Supporting Information

S1 Appendix Questions and Confederates’ Answers for RCIT (DOC)

S1 Audio Disclosure segment–Intimate–male actor. (M4A)

S2 Audio Disclosure segment–Intimate–female actor. (M4A)

S3 Audio Disclosure segment–Casual–male actor. (M4A)

S4 Audio Disclosure segment–Casual–female actor. (M4A)
