# Correction: When "In Your Face" Is Not Out of Place: The Effect of Timing of Disclosure of a Same-Sex Dating Partner under Conditions of Contact

**DOI:** 10.1371/journal.pone.0138937

**Published:** 2015-09-18

**Authors:** Sharon K. Dane, Barbara M. Masser, Geoff MacDonald, Julie M. Duck

There is an error in reference 71. The correct reference is: Abele, AE. The dynamics of masculine-agentic and feminine-communal traits: Findings from a prospective study. Journal of Personality and Social Psychology. 2003; 85: 768–776.
