# Supplementary material for: When "In Your Face" Is Not Out of Place: The Effect of Timing of Disclosure of a Same-Sex Dating Partner under Conditions of Contact
Source: PLoS One. 2015 Aug 26;10(8):e0135023. doi: 10.1371/journal.pone.0135023 (PMC4550461; doi:10.1371/journal.pone.0135023)
Supplement: S1 Appendix — (DOC) [file pone.0135023.s001.doc]

**Appendix**

**21 Questions and Confederates (male and female) Responses for the RCIT (Studies 1 and 2)**

**2 Timing (early vs. delayed) x Context (casual vs. intimate)**

**Participants were advised that the filmed confederates had 30 minutes to look at half of the questions to help prepare their answers. They were informed that the remaining questions were new to them to provide some spontaneity. (…. = were confederate pauses slightly). Note: the confederate tells both the cooking story and the brining dating partner home to family story but only discloses in one of them.**

1. **Describe the last pet you owned.**

Well I guess you could call this a pet. I had this tiny black mouse which I had to keep in the garage….because we weren’t allowed pets in the flat we were renting. It ran away as I was cleaning the cage and I never saw it again. I think I only had him for about 4 months.

1. **Describe one funny or embarrassing think that happened to you at school.**

My friends never let me forget this one! I was in year 10 and we had a sports uniform that was a yellow polo shirt and green shorts. One day I spilt my shorts in class. Bending over or snagging them on something…. Anyway…I had to wear them for the rest of the day with red underpants poking out of green shorts. I was called “Christmas Ass” for a few days after that! Some of my friends still call me that!

1. **What is one of your favourite pastimes and why?**

Swimming …ah no actually I really like to go for a long walk….. Not necessarily for exercise, and totally irregularly…. I love to just take off and roam for a couple of hours. I like reflecting....at least when I get the time.

1. **INSERT EARLY DISCLOSURE** – Casual Story - Cooking Experience Q**. Do you remember having a disastrous cooking experience? If so, what was it?**

I had these friends over for dinner and decided it was my turn to do the cooking, so I sent my partner….(disclosure – this guy/girl, no disclosure) that I was dating at the time off to buy the food. They (disclosure - he/she) came back with this really expensive cut of beef… So I made a really tricky beef dish…and I was pretty proud of myself… but then I found out that two of my guests were vegetarians!”

OR Intimate Story Visit Home with Partner Q. **Has your family ever disapproved of any of your dating partners? If so, explain.**

I remember the first time I brought home someone (disclosure - this girl/guy) I was interested in at the time. Some of my family seemed cool but I remember it was difficult ….because I knew that the others really disapproved (disclosure - of her/him)…. It caused a bit of a clash

1. **Give an example of a happy memory from your childhood.**

I remember this swimming hole near a beach my family would always go to…somewhere up the coast…. It had a giant rope swing and it was really deep…. My brother, sister and I loved going there…better than the beach…. I’d like to go back one day…if I can find it!

1. **If you could invent a new flavoured ice-cream. What would it be?**

Gee…I think they have invented every flavour by now!......maybe tim tam…..but that’s pretty close to cookies and cream(looking upward thinking)…..maybe some sort of savoury flavour for a change….(pull face)hmm maybe not…..oh I don’t know! ..next question

1. **What if anything is too serious to be joked about?**

I guess some people turn anything into a joke….I think it’s ok to joke about your own serious issues…. but I don’t think its right to joke about other peoples problems, especially if they are serious to them. I mean little things with friends are ok but you need to know when and where to draw the line. I know the newspapers sometimes make a joke about some really sensitive issues…sometimes I find this inappropriate.

1. **Do you prefer digital watches, or analogue? Why?**

(Look happily surprised by the questions) “Hey is this a trick question?” Well…I guess I would have to say analogue…(shrug) only because I think some of the better looking watches come in analogue

1. **What is one of your favourite types of food?**

Ummm……good pizza….ah no actually… I have a recent craving for Indian food…but only the real genuine stuff.

1. **If there was one thing you could save in a fire, other than your loved ones, pets, or pictures of them, what would it be?**

Umm, my guitar…..I think….I’ve grown attached to it and its worth a bit as well…so yeah I think I would save that.

**11)** **If you could spend a day with a famous person dead or alive who would this be and why?**

Well there are probably many……(long think)…I just can’t think of one off the top of my head…..(think) nope….. (leave unanswered and go onto next question)

**12)** **Give an example of something you regret doing?**

Not keeping in contact with a good friend. I moved away and then time just slipped by. I kept thinking I really should call or write but never got around to it. Now I don’t even know where to make contact….they’ve moved.

**13)** **If you were given money to redecorate your kitchen, what colours and style do you think you would choose?**

I think I’d go for the white and stainless steel look… a bit like the IKEA magazine …It would need one of those separate benches in the middle with plenty of bench space around it…..and all the cooking appliances and stuff at the walls around the edges….This way more than one person could cook at a time!

1. **Give one example of an adventure trip or holiday that went wrong.**

I went to Bunya Mountains with friends for a few days…. but just before we left I had an argument with one friend….I think it all started over a lost CD which seems really stupid now…..anyway we spent the whole trip making backhanded comments to each other ……which ended up ruining the whole trip.

1. **Where would you like to travel and why?**

I would love to go and live in France for a while…..I’ve learnt quite a bit about their culture and I’ve been almost fluent in the language…..so I’d love to get a good hold of the language at last…..and just enjoy all the stuff you hear about on a day to day basis.

1. **What abilities or qualities would you look for in a romantic partner?**

Umm…someone who is sure about themselves…has a passion for life…you know a kind of sense of determination and a zest for life…..Oh and obviously someone who is honest…..not just honest with me but with themselves as well…no pretenses

**17) What was your high-school like?**

Okay I guess…good opportunities and great equipment….. People were generally okay…….I guess like most schools it had it pluses and minuses.

**18) If you could change anything about the way you were raised what would it be?**

That’s hard…..well I know I would at least change dinner time to have been later

…like once the sun had gone down…. It drove me crazy how early we all ate…I’d still be full from snacking after school and wouldn’t eat much dinner…but than I’d get hungry later…I never got used to it!

**19) What is it about friendship you most value and why?**

…..(think)….well I guess that the pressure is off. They are not teachers or parents but

someone you can just relax and laugh with. You don’t feel you have to be a certain “someone” or act a certain way…you can just be you. At least in the case of good friends.

**20) INSERT DELAYED DISCLOSURE** – Casual Story - Cooking Experience Q**. Do you remember having a disastrous cooking experience? If so, what was it?**

I had these friends over for dinner and decided it was my turn to do the cooking, so I sent my partner….(disclosure – this guy/girl, no disclosure) that I was dating at the time off to buy the food. They (disclosure - he/she) came back with this really expensive cut of beef… So I made a really tricky beef dish…and I was pretty proud of myself… but then I found out that two of my guests were vegetarians!”

OR Intimate Story Visit Home with Partner Q. **Has your family ever disapproved of any of your dating partners? If so, explain.**

I remember the first time I brought home someone (disclosure - this girl/guy) I was interested in at the time. Some of my family seemed cool but I remember it was difficult ….because I knew that the others really disapproved (disclosure - of her/him)…. It caused a bit of a clash.

1. **If you could be granted one magic wish what type of quality or ability would**

**ask for?**

Ahh…..that’s easy…..to fly. I often have dreams that I’m flying…sort of like swimming through the air and looking down. Sometimes it’s a bit disappointing waking up and realizing up I’m stuck with walking!
